# The British Hospitals' Association Conference

**Published:** 1911-06-10

**Authors:** 


### THE BRITISH HOSPITALS' ASSOCIATION CONFERENCE.

### A SPECIAL COMMITTEE TO CONSIDER THE BILL.

THE Board-room at St. George's Hospital, which was very kindly placed at the disposal of the British Hospitals' Association on Friday last week, was packed with hospital representatives from all parts of the kingdom, many of whom had travelled up at short notice to take part in the Association's conference on the Insurance Bill. A glance at the full list of those present, which we publish below, shows that over two hundred hospitals were represented. Punctually at three o'clock, Mr. A. W. West, one of the hon. secretaries of the Association and treasurer of St. George's Hospital, was voted to the chair, and proposed that the Insurance Bill be so amended that the usefulness of the voluntary hospitals should remain unimpaired, and that a sub-committee of eighteen, with power to add to its number, should be appointed to wait upon the Chancellor of the Exchequer and to place before him such facts as will lead to the consideration of the position of the hospitals. He suggested that if the State made some contribution, they must expect, and certainly would not fear, State inspection and audit of their accounts. Sir William Cobbett, chairman of the Manchester Royal Infirmary, in seconding, remarked that while the finances of the voluntary hospitals would be affected by the Bill most seriously, the most important part of their work, the treatment of in-patients, would not be diminished. The compulsory contribution of employers would make it impossible for hospitals to ask them for a continuance, much more for an increase, of their subscriptions. The treasurer of Leeds General Infirmary, Mr. Charles Lupton, copies of whose concise summary of the Bill had been handed to many of the audience, followed, and while repudiating direct subvention from the State, said that the hospitals would be on far less dangerous ground in accepting payment for work actually done. Mr. C. S. Loch, secretary of the Charity Organisation Society, urged the importance of maintaining the self-governing principle in hospitals, which should be treated as co-operative colleagues, and not as mere official entities, under the scheme. More than one practitioner then addressed the meeting, and the discussion threatened to be dissipated on to side-issues till Mr. Roberts, of St. Thomas' Hospital; Mr. A. W. Davis, secretary of the Hospital Saturday Fund; and Lord Inverclyde once more recalled it to the main issue. The motion for the appointment of the sub-committee to wait on the Chancellor created some opposition until the chairman made it clear that no propositions would be addressed to him till the hospitals had had another opportunity of considering them. Votes of thanks to St. George's Hospital for its hospitality, and to Mr. West for presiding, were then carried, and the assembly dispersed in the expectation of a further meeting to consider the actual propositions which its sub-committee will recommend to Mr. Lloyd George.

### FULL REPORT OF SPEECHES AND RESOLUTIONS.

A SPECIAL meeting, convened by the British Hospitals Association, was held in the Board-room of St. George's Hospital on Friday, June 2, to consider the Government's Insurance Bill, and the attitude of hospitals in regard to it. The meeting elected Mr. A. William West, Chairman of St. George's Hospital, to preside.

There were present :—

Sir E. Hay Currie, Hospital Sunday Fund.
Mr. C. W. Thies, Royal Free Hospital, W.C.
Mr. P. Michelli, Seamen's Hospital, Greenwich.
Mr. A. W. Davis, Hospital Saturday Fund.
Mr. J. H. Cooke, Albert Infirmary, Winsford.
Mr. A. Watts, Queen Charlotte's Hospital, N.W.
Mr. R. J. Coles, Addenbrooke Hospital, Cambridge.
Mr. R. J. Johnstone, Royal Victoria Hospital, Belfast.
Mr. H. Eason, Guy's Hospital, S.E.
Mr. H. Marsh, Addenbrooke Hospital, Cambridge.
Mr. W. H. Pearce, Children's Hospital, Paddington Green.
Mr. T. H. F. Lapthorn, Royal Portsmouth Hospital.
Mr. B. Wagstaff, Royal Portsmouth Hospital.
Mr. F. W. Ray, Wolverhampton General Hospital.
Mr. A. R. Gray, Royal Infirmary, Aberdeen.
Dr. Goff, Western Infirmary, Glasgow.
Mr. H. Edmonds, Homœopathic Hospital, London.
Sir Matthew Arthur, Bart., Western Infirmary, Glasgow.
Sir W. Cobbett, Manchester Royal Infirmary.
Dr. Robertson, Victoria Infirmary, Glasgow.
Mr. A. Langton, Royal Free Hospital, W.C.
Mr. W. T. Partrick, Royal Surrey County Hospital, Guildford.
Mr. F. W. Drewett, Prince of Wales's Hospital, Tottenham.
Sir George Beatson, Glasgow Western Infirmary.
Mr. W. Pearson, Gravesend Hospital.
Mr. Fred Smith, Warneford Hospital, Leamington.
Mr. T. Hayes, St. Bartholomew's Hospital, E.C.
Mr. W. E. Lister, Royal Infirmary, Doncaster.
Mr. W. Ashe, Evelina Hospital, S.E.
Mr. R. Thorp, St. George's Hospital, S.W.
Mr. F. Messent, E. S. and Ipswich Hospital.
Mr. H. B. Dickinson, Essex County Hospital.
Mr. G. Roberts, St. Thomas's Hospital, S.E.
Colonel Harding, Addenbrooke Hospital, Cambridge.
Mr. Stewart Johnson, Hospital for Sick Children, W.C.
Mr. G. Ruddle, Salford Royal Hospital.
Mr. R. Knowles, Derbyshire Royal Infirmary.
Dr. W. Harris, St. Mary's Hospital.
Mr. R. Johnson, University College Hospital, W.C.
Mr. J. C. Buchanan, Metropolitan Hospital, N.E.
Mr. E. W. Morris, London Hospital, E.
Mr. H. C. Barker, London Hospital, E.
Colonel Broadbent, Royal Berks Hospital, Reading.
Dr. Mackintosh, Western Infirmary, Glasgow.
Mr. J. H. Shaw, Southport Infirmary.
Mr. Howard J. Collins, General Hospital, Birmingham.
Mr. G. E. Aspinall, Halifax Royal Infirmary.
Dr. Fraser, Royal Infirmary, Dundee.
Mr. W. A. Tait, Royal Infirmary, Edinburgh.
Mr. F. S. Goodwin, Birmingham General Hospital.
Captain Crosfield, Warrington Infirmary.
Mr. George Priestman, Royal Infirmary, Bradford.
Mr. Charles Lupton, General Infirmary, Leeds.
Mr. Albert Coy, Warneford Hospital, Leamington.
Mr. J. W. Aldous, East Suffolk and Ipswich Hospital.
Mr. A. Griffiths, East Suffolk and Ipswich Hospital.

Mr. J. A. Rudd, Coventry and Warwichshire Hospital.
Mr. E. Forster, Derbyshire Royal Infirmary, Derby.
Mr. C. S. Loch, Charity Organisation Society.
Mr. Arthur Moore, County Hospital, Lincoln.
Mr. C. A. Mason, Birmingham and Midland Eye Hospital.
Mr. W. Austin Leigh, St. Mary's Hospital.
The Lord Inverclyde, Western Infirmary, Glasgow.
Mr. James Macfarlane, Royal Infirmary, Glasgow.
Dr. Macgregor, Victoria Infirmary, Glasgow.
Mr. J. M. Thorn, Royal Infirmary, Glasgow.
Mr. R. H. Wilson, Charing Cross Hospital, W.C.
Mr. Albert Hastings, Children's Hospital, Birmingham.
Mr. Walter Alvey, Charing Cross Hospital, W.C.
Mr. H. S. Tannard, King's College Hospital, W.C.
Mr. Walter Carnt, Royal Infirmary, Manchester.
Mr. F. Wood, Brompton Hospital, S.W.
Mr. T. Ryan, St. Mary's Hospital, W.
Mr. C. E. Barnes, Chesterfield and N. Derbyshire Hospital.
Mr. Charles Hyde, Queen's Hospital, Birmingham.
Dr. J. Morton, Mount Vernon Hospital, N.W.
Major Christie, London Fever Hospital.
Mr. G. A. McDowall, St. Mary's Hospital, Plaistow.
Mr. S. M. Guennell, Westminster Hospital, S.W.
Mr. A. Franklyn, Royal Waterloo Hospital, S.E.
Mr. L. H. Glenton-Kerr, Gt. Northern Central Hospital, N.
Mr. Warrington Haward, St. George's Hospital.
Mr. L. H. M. Dick, Royal Nurses' Pension Fund.
Mr. F. B. Lewis, Buchanan Hospital, St. Leonards-on-Sea.
Mr. Harry A. Bone, Miller General Hospital, Greenwich.
Mr. E. P. Crowley, Faringdon Cottage Hospital.
Mr. E. D. Macnamara, West End Hospital for Nervous Diseases, W.
Mr. B. W. Ford, Children's Hospital, Southampton.
Mrs. Westlake, New Hospital for Women, London.
Dr. Nathan, Invalid Children's Aid Association.
Mr. G. H. Hamilton, National Hospital, Queen Square, W.C.
Mr. G. T. Bratson, Glasgow Cancer Hospital.
Mr. Thomas Kay, Stockport Hospital.
Mr. F. Tunbridge, Stockport Hospital.
Mr. James Penny, Enfield Cottage Hospital.
Mr. Walter Sleape, Southwold Cottage Hospital.
Mr. J. H. Goudie, Ayr County Hospital.
Mr. A. S. Wills, Bath Royal Mineral Water Hospital.
Dr. Livingstone, Dumfries and Galloway Royal Infirmary.
Mr. G. Martyr, Royal Victoria Hospital, Bournemouth.
Mr. H. W. Ewen, Cottage Hospital, East Cowes.
Mr. C. S. Dickens, Royal Sussex County Hospital.
Mr. H. R. S. Druce, Central London Ophthalmic Hospital, W.C.
Mr. T. S. P. Gregory, Grantham Hospital.
Mr. R. Orde, Royal Victoria Infirmary, Newcastle.
Dr. Thomas, Newport and Monmouthshire Hospital.
Mr. R. V. Hearn, Wimbledon Cottage Hospital.
Mr. George Dineen, Passmore Edwards Hospital, Willesden.
Dr. Kingsford, Passmore Edwards Hospital, Willesden.
Mr. M. W. Spruce, G.W.R. Medical Fund, Swindon.
Mr. M. H. Munro, G.W.R. Medical Fund, Swindon.
Mr. J. G. Gillett, Cottage Hospital, Faversham.
Mr. R. H. P. Walker, Cottage Hospital, Walton-on-Thames.
Dr. Drabble, Cottage Hospital, Walton-on-Thames.
Mr. H. Barney, Royal Berks Hospital, Reading.
Mr. T. G. Adnitt, Northampton Hospital.
Mr. J. G. T. Buckle, University College Hospital, W.C.
Mr. W. Wren, Alexandra Hospital, Aldershot.
Mr. J. Frances, City of London Lying-in Hospital, E.C.
Mr. S. W. Lacey, East End Mothers' Lying-in Hospital, E.
Mr. W. Gould, Mildmay Mission Hospital, E.
Mr. Thomas Maddock, Wellingborough Cottage Hospital.
Mr. J. A. Cowley, Victoria Infirmary, Northwich.
Mr. J. R. Motion, Poor Law Institution, Glasgow.
Mr. R. Lucas, Middlesex Hospital, W.
Mr. R. Kershaw, Central Throat and Ear Hospital, Gray's Inn Road.
Mr. A. E. Gaddum, Manchester Royal Eye Hospital.
Mrs. Rose, Edinburgh Hospital for Women.
Dr. Bullock, Eversfield Hospital, St. Leonards-on-Sea.
Mr. J. G. Thornton, Clevedon Cottage Hospital.
Mr. J. Nichols, General Hospital, Dudley.
Mr. J. J. Webb, General Hospital, Tunbridge Wells.
Dr. R. Clegg, Victoria Hospital, Accrington.
Mr. W. C. Douglas, Potters Bar Cottage Hospital.
Mr. J. M. Haslip, St. John's Hospital, London.
Mr. F. H. Moore, Borough Hospital, Birkenhead.
Mr. F. H. Moore, Royal Infirmary, Liverpool.
Major Tyler, Stockport Infirmary, Stockport.
Major Tyler, Manchester and Salford Hospital for Skin Diseases.
Mr. B. S. Briggs, Clayton Hospital, Wakefield.
Rev. J. E. Samuel, Blackburn and E. Lancashire Infirmary.
Mr. J. Kennett, Royal Victoria Hospital, Folkestone.
Mr. Benjamin Brooks, Hull Royal Infirmary.
Mr. J. F. Bache, West Bromwich District Hospital.
Mr. John Wood, Burton-on-Trent General Infirmary.
Mr. W. J. P. Marling, Stroud General Hospital.
Mr. Hy. Toulmin, St. Albans and Mid Herts Hospital.
Mr. Frederick Gore, Margate Cottage Hospital.
Mr. Stanley Wilkins, Royal Bucks Hospital, Aylesbury.
Dr. Basil Rhodes, North Staffs Infirmary, Stoke-on-Trent.
Mr. Jas. Smethurst, Warrington Hospital.
Mr. G. A. Bond, Barnsley.
Mr. F. A. Woodcock, Bury Infirmary.
Mr. James Barker, Ebbw Vale Hospital.
Mr. Thomas J. Rees, Ebbw Vale Hospital.
Mr. Edward Hemingway, Dewsbury Infirmary.
Mr. J. F. Wintringham, Grimsby and District Hospital.
Mr. Edgar Fitton, Dewsbury Infirmary.
Mr. E. Sharwood Smith, Newbury District Hospital.
Mr. Francis Nunn, Colwyn Bay Cottage Hospital.
Mr. W. H. Whitfield, Alexandra Hospital, Queen Sq., W.C.
Lieut.-Colonel R. D. Garnons Wilson, Brecon County and Borough Infirmary.
Mr. C. Postgate, North Riding Infirmary, Middlesbrough.
Mr. H. F. Ransome, Altrincham Hospital.
Rev. R. B. Gwyder, Swansea General Hospital.
Mr. E. C. Smith, Middlesbrough.
Mr. W. M. Philips, Manchester Northern Hospital.
Mr. A. D. White, Royal Hampshire County Hospital.
Dr. Davy, Royal Devon and Exeter Hospital.
Mr. B. Hobbis, Maidenhead Cottage Hospital.
Mr. H. Little, Hanwell Cottage Hospital.
Mr. L. Tod, Bridgnorth and South Shropshire Infirmary.

### Why the Meeting was Convened.

The Chairman, after acknowledging the honour done to St. George's Hospital by inviting him to preside, said the object of the meeting was as follows: The British Hospitals Association, in considering the possible effect of the National Insurance Bill from the point of view of the voluntary hospitals, came to the conclusion that it would be a good thing to call a representative meeting of those connected with the voluntary hospitals. It was felt that that was the only organisation in existence which had the power to call such a meeting. That the decision to do so was the right one was evidenced by the fact that there were present representatives from over 200 voluntary hospitals in the country, many of whom had been travelling all night in order to attend. On behalf of the Association he wished to state that any measure which tried to improve the condition of the health of the nation must meet with its entire sympathy, and that any difference of view which might be manifest must be on matters of detail, not of principle. Whatever individual opinions might be as to whether the Bill was a good one or not, of one thing he was sure, as all would be, that the effect of the Bill must be to alter profoundly the whole system of the voluntary hospitals as it was now known. Many of those present would feel that, in the first place, since all the nurses, servants, and other employees of the hospitals, had to be insured by the hospital, they had to pay their own contribution towards the insurance, and so long as they were resident in the hospital they had always in the past been treated as in-patients. Thus they would never qualify for the State benefits. Some of those present felt that this was a hardship, which would mean a tax on the hospitals of something like £20,000 to £30,000 per annum to pay even the insurance premiums. It was strongly felt that the nurses and servants in the hospitals should be exempted. Under clause 12 of the Act, in its present form —although he believed a modification had been suggested by Mr. Lloyd George—there was a special proviso which exempted cases of sickness, disablement, or maternity benefits from making a contribution towards the hospital funds, the whole benefit going towards the maintenance of the family. He wished to submit some figures in that connection. Taking the year 1909, of thirty-one hospitals with medical schools in England, Scotland, and Ireland, eighty-two general hospitals, and forty-nine special hospitals (excluding consumption hospitals), the total income from all sources amounted to £2,617,648, the total expenditure being £2,406,911. On the new scheme of national insurance it might be argued that, although there would be a large class which would require assistance from the charitable public, such as the wives and families of those who were exempted from the scheme, those whose income was over £160 a year, which, from one cause or another might have suddenly ceased, and of those who for some reason had omitted to keep up their payments, the vast majority of the public, feeling that they were paying to a national insurance scheme, would no longer see the necessity of contributing to the hospitals as in the past. That could be said of donations and legacies. All legacies would probably cease, and the Hospital Saturday Fund and Hospital Sunday Fund collections would probably come to an end to a large extent. The ordinary subscriptions to the hospitals in the year 1905 amounted to £327,924, and ordinary donations £269,498, while legacies amounted to £492,601; the Hospital Saturday Fund and workmen's collections, £212,496; patient's payments, £71,706; special donations, £486,390. That gave a total of just under £2,000,000. That left £496,000 as the only assured incomes to maintain those hospitals. Some of those present did not understand why an assured person meeting with an accident in the street and being removed to a hospital was to make no contribution to that hospital, nor others on his behalf. There appeared to be no provision made by the State for cases of operation. So far as he was able to gather at present, the Bill provided for the ordinary everyday ailments of life for one section of the community only, and made no provision for more serious and expensive cases, even among the insured.

### A Deputation to Mr. George.

He could briefly state that the idea of the meeting that day was to pass, if approved, some resolutions with the object of expressing the view of the meeting on the Bill, and appointing a committee to wait upon the Chancellor of the Exchequer in order to submit to him the whole case of the hospitals, and arrive at some definite arrangement. The suggestion which was in the minds of those who arranged the meeting—though they were waiting to hear what others had to say on the subject—was that some form of contribution towards the hospitals should be made in some way or other. Whether it should be made by the State as *per capita* grant, or a grant, as in many of the Colonies, where the State contributed a sovereign to every sovereign collected by the hospitals themselves, he could not say. But there could be little doubt that if once the system were admitted into hospitals, upon some definite payment, it would be agreed that there should be State inspection and State auditing of accounts. He doubted whether any hospital in the kingdom would mind that. The hospitals carried on their work in the open, and they had nothing to conceal, and he did not think it likely that anyone would object to any proper examination by proper people, who would inspect the method of administration of hospitals and see that their accounts were kept on some agreed basis. In London that was practically done now. There was the King's Hospital Fund, which sent round its inspectors, who notified in what form they required the accounts to be kept; there was also the organisation of the Saturday Fund, as well as that of the Sunday Fund. Nothing but sympathy and help were derived from those inspections, and if the Government sent inspectors, sympathy would no doubt be forthcoming from them. He did not think that was a danger. But if there was State aid there must be State inspection. If it could be shown that the Bill would benefit the vast numbers who in the future would use the general hospitals, the managers of the hospitals would welcome the National Insurance Bill and do all they could to help matters forward. He was only expressing the general opinion when he declared that some change was desirable in the Bill before it became law. One of the proposals was that representations should be made to the Chancellor of the Exchequer with that end in view.

### The Resolution.

He proposed to submit the following resolution to the meeting for its acceptance or to be dealt with as those present might see fit. He would read its terms at this stage, as it was desirable that the speeches should be directed to something quite tangible :—

"That this meeting of the managers and administrators of voluntary hospitals, convened by the British Hospitals Association, welcomes any legislation which, by a reasonable arrangement, will benefit the sick poor and continue to make use of the machinery of voluntary hospitals, and hopes that the Bill will be so amended that their usefulness may be continued and their efficiency remain unimpaired."

He suggested that should the first part of the resolution. Another suggested clause was :—

"That the British Hospitals Association be requested to appoint a sub-committee of eighteen, the Honorary Treasurer and the two Honorary Secretaries being *ex-officio* members, with power to add to their number. That such committee be authorised to wait upon the Chancellor of the Exchequer to draw up and place before him such statement and particulars as it thinks advisable with the view to the position of the voluntary hospitals in the future being considered, and to suggest amendments, or to take such other steps as it may from time to time think necessary."

At a later stage the names of certain gentlemen would be proposed to constitute that committee. He called upon Sir William Cobbett, Chairman of the Manchester Royal Infirmary, to second the resolution.

### The Effect of the Bill on Hospitals.

Sir William Cobbett said they were met that afternoon to see what steps could be devised for the protection of the interests of the hospitals which those present represented, with reference to the State Insurance Bill. He believed—though he did not know whether others in the room would agree with him—that there was among the managers and administrators of hospitals a well-grounded anticipation that if the State Insurance Bill passed in its present form it would have a most injurious effect upon the financial condition of all hospitals. (Hear, hear.) Therefore, it was necessary that those concerned with the hospitals should take some prompt, business-like, and strenuous action to put before the Minister in charge of the Bill the views which they held on the subject, and the suggestions which they had to submit to him for the guarding of their interests, and, through those interests, the interests of the sick and suffering poor. The effect that the Bill was likely to have upon hospitals would be conveniently dealt with by considering it under two heads: The effect that it would have upon the work of hospitals, and, secondly, the effect it would have upon their funds. So far as he had been able to gather from the communications he had had from others who were in a like position to himself with reference to hospitals, they seemed to be agreed that the Bill would have no effect in the reduction of that which was the most onerous, most extensive, and most important work of the hospitals. That was what he might describe as the in-patient work. Nobody seemed to believe that the number of in-patients or the costs of carrying on the hospital for their benefit would in any way be affected by the Bill, and so far as the needs of in-patients were concerned, the necessity for the continued existence of hospitals and for the expenditure of funds as great as, or even greater than, now, would still continue.

### Out-Patients' Departments.

With regard to the out-patients' department, there seemed to be some difference of opinions; but most people appeared to think that the work of the out-patients' departments would be reduced. *Prima facie*, it would certainly seem that that was because the medical benefit which was conferred free by the Act of Parliament was precisely the benefit which the out-patient department of a hospital conferred free, namely, advice and medicine. Yet it did not seem to be certain that the work of the out-patient department would be affected to such an extent that it would be unnecessary for out-patient departments to continue to exist. Many, particularly among the medical men on the honorary staff of hospitals, thought that the effect would be that the work would be very much reduced, and that the out-patient department would be a place to which patients would come only when a second opinion was required, or for purposes of consultation between medical men; and that in this way, while the work would be greatly reduced, the department in some shape or other would be still a necessity. He ventured to put forward that view for himself. If that were to be the result, he feared that the out-patient departments of hospitals would lose in popular favour, that they would cease to be considered in the same way as they were now by the general public, particularly workpeople, as a place of resort for all classes who were under the necessity of going to such places whenever they had ailments which they thought could be better treated there than elsewhere. And the moment that department commenced to suffer in popular estimation the effect would be felt in the decrease of subscriptions and donations to the hospital. But when those observations had been made he did not think that the Bill would have any great effect upon the work of hospitals. There would still be the main body of work to be dealt with by them, and the same, or even a greater, expenditure would be required.

### The Financial Question

His other heading was, the way in which the Bill would affect the funds of hospitals, and that presented a formidable question. The tendency of the Bill—he did not say that was its object—was that it would not have the effect many thought it would. The tendency was to divert at its source the flow of subscriptions and donations which had hitherto reached the coffers of hospitals. (Hear, hear.) Speaking for his own district, on the ground of his familiarity with it, he could say that the main contributors, in the way of donations and subscriptions to the annual income of the Manchester Hospital, were derived from two classes: the employers on the one hand, and the employed on the other. The Bill provided for taxation from both sources for the very kind of relief which was now afforded them in the hospitals, or at any rate in part. One very well-known company, a director of which was a member of the Board of the Manchester Infirmary, which company had always been most liberal and generous supporters of that hospital, would have to pay as the employers' contribution under the Act £19,000 per annum. He was told that by the director in question. If that was to be the new condition, with what face could one go to an employer who was contributing for sick benefit and for free medical relief £19,000 per annum and ask him for an annual subscription to one's hospital? In Manchester there were a large number of contributors who were employers of quite a different class, a much humbler class, and the Manchester Infirmary received from them a large sum of money in amounts of one guinea or two guineas, or three guineas; and he confessed that he looked forward with great apprehension to the effect which the Bill would have upon these small employers. If it should have the effect upon them which he feared, he could not help thinking that the effect on the funds of his hospital would be very serious indeed. But he thought there were many suggestions which might be made for the protection of the hospitals and for the improvement of the provisions of the Bill with reference to them. It would take too long to discuss those matters in detail, nor, indeed, was that the place, for it could be done, and only effectively done, by a comparatively small number of men sitting together. It was for that reason he seconded the resolution authorising the appointment of a committee to consider the details and to interview those in charge of the Bill, to suggest amendments, and at the same time to keep itself, as far as possible, in touch with the various provincial hospitals. That seemed to be the only wise and business-like course to take. It was one which should be taken promptly, because he noticed by to-day's papers that the Chancellor of the Exchequer said he anticipated that he would get the Bill through in August. That made it clear there was no time to lose, and that the Bill would soon be committed. Bearing in mind the holidays which would intervene, it was desirable that the committee should be appointed at once and get to work, and should represent the interests of the hospitals from a business point of view and strenuously to the Chancellor of the Exchequer, and as soon as might be.

### The Sources of Hospital Income.

The Chairman asked whether there were any amendments. As there was no response, he said he would call upon Mr. Charles Lupton, Treasurer of the Leeds General Infirmary. That gentleman had issued and circulated a printed paper on the subject, and it was a contribution such as they had been looking for for some time. It put the matter clearly and concisely, and Mr. Lupton's views would be interesting to hear.

Mr. Charles Lupton said he did not propose, in view of the paper which had been mentioned, to go through the arguments on the question; moreover, what he had intended to say had been much better put by the Chairman. It appeared to him that in framing the Bill the Chancellor of the Exchequer, for some reason, had not put himself in close connection with the managers of such hospitals as were mainly maintained by charitable contributions. There were certain great hospitals in London which were so fortunate as to have an income of their own, quite apart from the money which they could obtain annually from the charitable public. Such hospitals as those were outside the sphere of any trouble which such a Bill as this could bring upon them. In England there was the extraordinary fact that the greatest work which had been done for the humbler classes in the matter of medical assistance had been done for centuries by hospitals which were essentially maintained by contributions which they got from day to day. In Leeds—and he thought their example was not different from others—they had £10,000 to £12,000 a year of income from endowment; and the rest of the expenditure, which was about £27,000 a year, was met from sources which could not be called certain from one year to another. That being so, it seemed to him essential that such hospitals as the Leeds one should take great care that in any new legislation which was brought forward the continuance of those subscriptions should not be jeopardised. He felt sure that the aims of those who constructed the Bill were very high aims indeed, and that they were actuated by pure philanthropy. But there was such a thing as philanthropy going astray —(hear, hear)—and he thought it was quite possible that in trying to cover a difficulty—which was, no doubt, a very great difficulty—and trying to cure an ill which was well-known to exist in our midst, there was some danger that the benefit aimed at might be largely done away with. Sir William Cobbett had expressed the view that, so far as in-patients in a large hospital were concerned, the Bill would make no difference whatever. In that he agreed with him entirely. All he would say in criticism of that was that an actual premium was offered by the Bill for people to become patients in the hospital, because as soon as they became patients in the hospital the contributions which they had paid for medical attendance would be diverted from medical attendance, and would be handed over to their dependants, if they had any. (Hear, hear.) Therefore, it was obvious that if a man was ill and wished to do the best he could for his family the best he could do was to gain admission to hospital, the hospital getting nothing.

### Out-Patients under the Bill.

In reference to out-patients, he did not take the same view as did Sir William Cobbett. There was no doubt, he thought, that the trivial out-patients, those who flocked to the hospital doors for very slight reasons indeed, could with good confidence have the hospital doors shut upon them; they could be told they were provided for otherwise, and could go to the doctor whose attention had been arranged for by the State. They could be told that they need not trouble to attend a hospital. But when one considered the matter, it was evident that the out-patient department was only partially occupied at present with that class of case. The other cases who came there were those who were recommended to come there, or sent by their own doctors; in many instances by club doctors, and sometimes from other classes. Some came in search of a second opinion. Some time ago, in his own hospital, they were endeavouring to meet the ever-recurring difficulties which arose with regard to out-patients. It was considered that those classes of persons should be treated as one, and that priority of attention should be given to people who were recommended to the hospitals by their own doctors, or were sent in by those doctors. And in the same class one ought to allow people to pass in who had been attending a doctor for a considerable time and who were still requiring further medical attendance. He believed it would be found that even if the out-patient work was limited to those two classes of people, the out-patient departments would be quite full of work.

### Special Departments and School Children.

All special departments came under those heads. One could not expect a general practitioner to have special knowledge of ophthalmology, nor to know all about ears and throats. And it was well known what an immense number of patients were sent to the hospitals in consequence of the recent medical inspection of schools. All those would still come, as would also patients requiring inspection and treatment by $x$-rays and other costly apparatus. He did not think there should be any delusion that any large number of patients would be lost to the hospitals as the result of the new Bill. When one looked at the Bill and considered the effect which it was going to have on finance, one was a little anxious. When an employer had to pay, in some cases 3d. and in some cases 4d., for the work which was set out in the Bill, and it was recognised that it was to supply ordinary treatment and attendance, including proper and sufficient medicines, treatment in sanatoria, and other institutions when suffering from tuberculosis or such other diseases as the Local Government Board, with the approval of the Treasury, might appoint for sanatorium benefit, it would be supposed that because those benefits were given, everything was given. Apart from the fact that there will be the sums given and which will appear in the accounts, they would believe that everything was done and there was no need to give. For several years a good deal of his time had been devoted to collecting money to run a hospital, and he knew the great difficulty there was in persuading the ordinary man of the necessity of the hospital. If one could get the layman to look at a hospital and take an intelligent interest in it the trouble was over, and money would be forthcoming. But he doubted whether there would be many visitors on those lines when such sums had to be paid as Sir William Cobbett had mentioned.

### How the Working Classes Support Hospitals.

When one turned to the case of the employed, the same remarks applied. In Newcastle at present he understood they got £17,000 a year towards the upkeep of their hospital from the working classes. In Leicester it was about £11,000, and in Leeds something over £7,000. He doubted whether much of that money was likely to be seen in the future. It would be difficult to show the differences there were between the two classes of persons among labouring people. In other words, there were only a certain number of people who would come within the benefits of the Bill at all. When one remembered the enormous sums which would have to be paid towards the expenses which the Bill contemplated, it did not seem likely that people would be anxious to remember that only about one-third of the working classes would receive any benefit from it at all. Wives were strictly cut off from benefit under the Bill during the period they remained as wives and did not become workers; and the children came into the same category. He hoped that in anything they did they would take great pains that people should not get a direct subvention from the State. He thought that would be a fatal mistake. It it could be arranged that the work done for the State should be properly paid for out of the fund, they would be on as nearly safe ground as they could be. But if a contribution was allowed to be made by the State of so many hundreds or so many thousands of pounds a year, according to the work done by the respective hospitals, the collectors might be dismissed, and one could cease trying to collect contributions from the charitable public. State aid meant the end of voluntary hospitals. (Hear, hear.) But if it were on the basis of work which the hospitals did, the ground was much less dangerous.

Dr. Garrod Thomas (Newport, Mon.) desired to call the attention of the meeting to the first page of the notes on the National Insurance Bill which had been circulated, and suggested the substitution of the expression "Diseases of an unusual type" for that used in the circular, i.e. "disease of a type not familiar to the general practitioner."

### A Plea for Exactness.

Mr. Cooke (Winsford) wished to say a few words, because those who had spoken so far represented large towns. His own town had only ten thousand inhabitants, and he was honorary secretary of the hospital, and had to do with the collection of money. He wished to speak also because, as a lawyer, he had to criticise the Finance Bill seriously. He wished the committee which would be appointed by this meeting to consider, first, they should not be rushed into any legislation; there should be plenty of time to consider it. And any words of the Chancellor of the Exchequer must be carefully weighed. He thoroughly supported the remarks of Sir William Cobbett, that to the country hospitals the Bill would mean that they would not get the subscriptions necessary for the support of the institutions. There was also the fact that the employer had to contribute, and a large amount had been instanced. As Clerk to the Justices, he often heard, when a person was summoned for the maintenance of relatives, the remark, "I have paid rates for twenty years; why should I now be called upon to support my father and mother?" Similarly, when this Bill became operative, would not the workman, when he had paid his contribution to the National Insurance Bill, say: "I am paying so much every week to the State, and therefore you have no right to ask me for a penny"? The same argument would be forthcoming from the workman as from the employer. He hoped, therefore, that any committee appointed would consider those matters carefully. The committee must know exactly what it proposed to put before the Chancellor of the Exchequer, and then place it before him in a polite manner.

### The Machinery of Insurance.

Mr. C. S. Loch (Charity Organisation Society) said it was very necessary to combine opinion on the question. It was desirable to reiterate what had been said by the seconder of the resolution, Mr. Lupton. The finances of the hospitals were placed in jeopardy by this Bill, as one could not reasonably doubt. No reference had yet been made to the parties with which the hospitals would have to deal if the Bill should pass. One party, he presumed, would represent the approved society, and that would be a party of insurers, backed by the central Government, by the Commissioners of Insurance. They might be interested in engaging on such terms as would strengthen their position, as carrying forward that National Insurance. Those terms might not be altogether compatible with what might be wished from the point of view of the hospitals. Therefore, in his opinion, it was very necessary that, if possible, the hospitals themselves should be represented on that body. Still more was that so in regard to health committees. They were formed in a very hybrid fashion, and they would have very great power indeed. They might wish all kinds of government to be impartially placed in their hands in connection with the transactions entered upon with the hospitals. Therefore he thought the position of the hospitals should be safeguarded in this respect, as well as the others which had been mentioned. He knew, from his interest in charities, that unless the charities were self-governing, interest would fall in regard to them. Quite apart from the financial question, they would go to the wall. (Hear, hear.) What was wanted was strong, self-governing, go-ahead institutions, trusted by the Government, and helping forward in that great design, as colleagues, and not merely as official entities. The other question he wished to press was what had been said already, but had not, he thought, been sufficiently emphasised. It was common, in all arrangements with Government Departments, to plan that the person who had assisted should be paid for by the Government Department, the voluntary institution which intervened to take care of the case being in that manner remunerated. There was a prospect of a good and useful method of self-government on that basis, the entire cost being paid for by the Government; because if an inadequate sum was paid, it might very well fall out that very great pressure might be put on the hospitals, and there might be a difficulty in keeping up the standard of work which they themselves desired. But above all it was necessary that the institutions dealt with in the Bill should remain self-governing charities, of good design, taking a definite share of the responsibility now being thrown on the country. In saying that, he did not lose sight of what had been said by previous speakers. He endorsed all that they said, and insisted on a very clear course being adopted if hospitals should undertake work on that new arrangement for the Insurance Commissioners.

### An Argument for Medical Committees.

Mr. Kay (Leamington) thought the meeting was missing an important point in the Bill, namely, Clause 14: "Every approved society and local health committee shall, for the purpose of administering medical benefit, make arrangements with duly-qualified medical practitioners for insurance purposes." The honorary officers and staffs of the hospitals, duly qualified practitioners, were the proper people to undertake that work. And if they could impress upon the Chancellor of the Exchequer the value of enabling their committees to be medical practitioners, much would be done to help the hospitals. He had much to do with workmen who were subscribing a penny a week for the benefit of the hospital, and he found that those men were in a great difficulty as to what was going to happen to them. They said that if there was one medical man to 3,000 persons, which he believed was the number reckoned, and some 1,500 men did not approve of that practitioner, what would they do? They must continue to go to that man. But if Mr. Lloyd George would grant the option of employing another medical man, the matter would be easier, and the working man would be helped. Working men required the best doctors, and they were very critical. For instance, in Leamington they had a first-rate oculist, yet many workmen who had trouble with their eyes said they wanted to go to Birmingham or some other large town for treatment. But under this Bill they must not do that: they would be obliged to go to the man who was appointed for them.

### A Medical Man's Views.

Dr. Basil Rhodes (North Staffordshire Infirmary) said he was sorry to intervene with a slightly discordant note, but he hoped it would not be thought that he was out of sympathy with the object of the meeting. It was mentioned by Mr. Lupton, as well as the last speaker, that some arrangements must be made about the medical man. Mr. Lupton seemed to think that some arrangement must be made with the Government to get paid for any work which was done by the hospitals. He, Dr. Rhodes, wished to utter a warning note, namely, that if such were done there would be trouble with the medical man. There should be careful consideration before that was arranged, otherwise it would defeat its own object.

He felt sure there would be no honorary officers at all, and one would be bound absolutely to the State hospital. Therefore he would like that point to be carefully considered by the committee in deciding in what way they would get remuneration or reward or return for the treatment. He thought that in regard to the suggestion of the last speaker that the Government should be asked to choose the doctors at the hospital, the same thing applied as he mentioned occurred at present, that probably men would not always want a hospital doctor to start with. He thought that often they did not. In any case probably the medical men would want more than that, that there should be a choice of doctors for the insured.

The Chairman remarked that matters of the kind just referred to would be taken into consideration later on by the committee which would be appointed.

### The Absence of Provision for Hospital Treatment.

Dr. Davy (Exeter and Devon Hospital) remarked that most of the speakers had omitted the fact that under the new scheme hospitals would have a much larger work to do than they had previously. As scientific knowledge increased, and the treatment of hospital patients became more scientific, so it would become increasingly necessary for insured persons to attend the hospitals for treatment. It was not a question of disparaging the general practitioner, but the surroundings of a hospital; the nursing and the scientific treatment after operations and after obscure medical cases, would come more and more into the forefront and they would have to be dealt with. He thought Mr. Lloyd George had forgotten that in his Bill, for he had made no provision by which those insured persons should be able to come to the hospital for treatment. If they did come to the hospital for treatment, some provision must be made to the hospital for the treatment. It should also be remembered that these insured people were coming to hospitals for treatment by highly skilled medical officers, and it was only fair that medical officers should have some remuneration; and they would cease, in a large number of cases, to be honorary medical officers on the same terms as at present. He hoped that point of view would be carefully considered.

### The Bill and the Great Funds.

Mr. Davis (Hospital Saturday Fund) said he wished to say a word on behalf of the Metropolitan Hospital Saturday Fund. That, as was known to all Londoners, was an Association for collecting contributions for the support of the voluntary hospitals of London, mainly from the working-classes. The effects of the proposed Insurance scheme would be very detrimental in some respects to the work of the Fund, and therefore of the voluntary hospitals of London generally. The contributions were obtained from the working-classes by means of a penny-a-week collection, a collection which was largely supplemented by employers of labour. The employers either gave the time and opportunities for the collections, or they gave 10 per cent., 25 per cent., or 100 per cent. to the contributions collected. His point was that if by the new scheme the employers as well as the employed were compelled to contribute to the State scheme, it followed, as night followed day, that the voluntary contributions from the same sources would in great measure be stopped; at least they would be seriously curtailed. Therefore it was very important that those interested in the voluntary hospitals of the country should meet, as was being done then, to bring forward their ideas in reference to the scheme, and place them before the Chancellor of the Exchequer, so that the interests of the voluntary hospitals might be duly safeguarded. With regard to the different benefits his ideas were rather nebulous at present, but with regard to the medical benefits he thought they would to some extent do away with the so-called abuse of the out-patient department. If properly qualified and experienced men who had been thoroughly trained at hospitals attended working-men at their homes there would be no need for them to go to a distant hospital. In regard to sanatorium benefit, he thought some such committee as that proposed would be able to place the claims of consumptives and others before Mr. Lloyd George, in order that diseases of that kind might be properly dealt with. If the health committees were properly instructed and empowered they would begin in the right place, at the home of the patient rather than elsewhere. He thought that the scheme which had recently been developed in London, and many years ago in Edinburgh, establishing anti-tuberculosis dispensaries, might well be considered by these health committees. He agreed that something should be done in the direction indicated by the motion before the meeting in order to have the claims of London and the country generally put before the Chancellor of the Exchequer.

### Another Plea for Exactness.

Mr. Messent (Ipswich Hospital) said he attended the meeting wondering what it was that was wanted, and hoping that he would be able to get some clear idea of what was being aimed at. At present he was as much in the dark as when they started. Before any deputation should attend before the Chancellor they should clearly understand what it was the meeting desired. The suggestion which had been made, and which looked as if it might be practical, was that for the work done by the hospitals for the insured patients a definite fee should be paid, or that the whole charge should be paid. That was all very well if it would satisfy the medical men. But if that payment was made, who was to take the fee? Or would the medical man do the work and take the fee? The request of the hospitals should be clearly formulated and put before the Chancellor so that he could be asked to embody it in his Bill.

### A Wail against the Wage Limit.

Dr. Robertson (Glasgow) remarked that for two days he had been listening to the speeches at the representative meeting of the British Medical Association and taking some part in that meeting. It could be clearly understood that when the State aid to hospitals began, from that very moment the question of charity went out of it—(hear, hear)—and when that happened the medical man employed on the staff of the State-aided hospital would demand not only to be paid, but to be paid well for the work he did. He therefore wished to emphasise the necessity of keeping up the voluntary character of the hospital, because that was the only way in which the hospitals could be managed through the country as before. If the medical man must be paid for the class of operation which were done, that would be a different thing from the six shillings per annum which Mr. Lloyd George was offering to the general practitioner. In the eyes of the doctors themselves general practitioners were to be badly used by the Bill. (Hear, hear.) So far as the medical men were concerned there was scarcely a good point in the Bill, and the doctors needed the sympathy not only of the general public but of the managers of the different hospitals. After a man had been twenty-five years practising, beginning in the lowest ranks of practice, he was seeing a better class of patient, and had gradually eliminated from his practice club patients. The bulk of patients he was seeing in his later years were those earning between £100 and £160 a year. And then Mr. Loyd George brought in a Bill which said that the patients from whom they had been having a fair fee, so that they could do with fewer patients, should come under the scheme, so that the practitioner had to start again and make a new practice at the rate of club patients. Medical men who had been many years in practice felt that prospect to be a very great hardship. Moreover they were not physically able to do such heavy work because of their age. He saw in a London paper that morning that doctors had been rather severely handled because they had put the question of money in the forefront of their deliberations, the question of sympathy having been put in the background. But the medical profession from time immemorial had never, in the eyes of the public, been wanting in sympathy. But when it came to a question of such an uncertain nature that it was not clear that the practitioner would be able to keep his wife and family after being so long in practice, the matter was different. The most important advice he had heard that day was that the Bill should not be rushed. (Hear, hear.) It was a shame that it should have been rushed as much as it had already. Why had the medical profession been neglected up to the last moment by Mr. Lloyd George? Why should he have gone to the Friendly Societies and taken all their advice and got their staff to help him to frame the Bill, while the medical profession, who alone were bound to carry it out, had not been consulted until now? Was that what one would expect from a statesman? Yet he had to thank Mr. Lloyd George, because this was the first occasion on which, to his knowledge, the medical profession had been unanimous on any question. (Loud laughter.) Their unanimity on this question could be found out, because he was satisfied that unless the Bill satisfied medical men, from the North of Scotland to the South of England they would have none of it; and how could such a scheme be carried out if the medical men said they would not do the work laid down for them in the Bill?

### State Intervention and State Management.

Mr. G. Q. Roberts (St. Thomas' Hospital) said he had no definite proposals to make to the meeting. When one looked into the details of the Bill, it filled one with dread as to the future. General practitioners had been very much before the public in the matter, and he thought, rightly. Hospitals scarcely existed, in the eyes of the public, at all. One important effect the Bill would have on hospitals was a financial one, and the second concerned their future government. There might be a few hospitals which might be in such a financial condition that they would be able to carry on in the future, but the mass of the hospitals of the country must continue to depend on purely voluntary ccontributions. The total number of people who were to be insured under the Bill was only 14,000,000, whereas the total population was 45,000,000 Was it to be concluded that the 31,000,000 were rich enough to pay for their own general practitioners? If not, how would they be attended to? It was known that only a small proportion of the 31,000,000 were able to pay for their own practitioner, a private medical man at home. But 14,000,000 did not represent the people for whom medical aid must be provided, but only a small proportion of that number. The hospitals treated cases brought to them which could not be referred to the Poor Law. All would agree that it was a matter of pride amongst Englishmen that when foreigners visited our hospitals, those who knew hospitals in other parts of the world and knew their working, said how highly satisfactory the voluntary hospitals of England were and how much they envied them. Much had been said about the German system of insurance, but it was the Germans who knew most about hospital work, and who appreciated most highly the voluntary methods in England. Mr. Lupton had very well said that if the hospitals did not get voluntary contributions, then the cases must be paid for. He did not see how they were going to get money assistance without some intervention in the management. That was nothing to the hospitals themselves; it was for the good of the poor people who came to hospitals; it was important for them that the administrative independence of the hospitals should be rigidly maintained. It was very well known how excellent was the voluntary aid given by the staffs of the various hospitals; they were the most able men who could be got, and candidates for the posts were those who had distinguished themselves in the work. In a general way, the governors of hospitals were able to choose the most able body of men available, and their expert services were entirely at the disposal of the poor patients who attended. But if there was to be State intervention and State management and State payment, that meant that those doctors were not going to work for nothing, but would require paying. And there would not be the same number on the staff; there would be a few who would be paid, and they would allow the work to devolute upon others. For the benefit of the patients what was needed was that there should be a continuance of the highly skilled staff which was the characteristic of the hospitals of to-day in this country. He wished to support very strongly the idea that a sub-committee be appointed to go into all the details, and he thought they should be authorised to make strong representations to the Chancellor of the Exchequer which would at least lead to a recognition of the magnificent work which has been done in the past by the voluntary hospitals.

The Secretary of the Association reminded the meeting that the Chancellor of the Exchequer admitted, when receiving the deputation of the British Medical Association, that the question was a very big and difficult one. That was a recognition which was very important.

### Further Discussion.

Mr. Smith (North Ormesby Hospital) suggested that the opinion of every hospital be asked, instead of taking only the decision of the 200 hospitals represented at the meeting. He made that suggestion before the resolution was put.

The Chairman said the suggestion of Mr. Smith had been carefully considered before the meeting, and, in view of the short time remaining, it was found impossible to carry it out. That would be realised when it was remembered that there were 800 hospitals concerned.

He read the resolution again, and was about to put it when

A question was asked whether it was proposed to send a deputation to the Chancellor of the Exchequer before another general meeting had been informed of the result of the committee's deliberations.

The Chairman replied that the general body would be asked to grant the committee power to do what they thought well.

Sir George Beatson (Glasgow Western Infirmary) asked whether it was certain that the hospitals represented would not be committed to any line of policy without their knowledge?

A gentleman present remarked that the hospitals would still be called upon to do the work, and no provision had been made to pay them. He hoped the committee would bear in mind the interests of the general medical practitioner, because it would be impossible for any sum to be taken out of the contributions towards the hospital upkeep. The committee should settle that question as to how the hospital treatment should be provided for without interfering or endangering the other matters of the Bill, which were separate altogether. He felt that the voluntary systems of the hospitals should be upheld.

The resolution was then put, and carried unanimously, amid cheers.

The Committee Appointed.

Lord Inverclyde said he came to listen to the discussion on the Bill in order that if it should reach the House to which he belonged he might have some knowledge of the opinions held by the Association, which was so well qualified to express its views. He had been asked to move suggested names to constitute the committee to consider the question, and to interview the Chancellor of the Exchequer. He hoped members of the Association would not be content with that, but would make the lives of the members of the House of Commons unhappy unless they fell in with their views. He suggested the following: Mr. Cosmo Bonsor, London; Sir William Cobbett, Manchester; Mr. Lupton, Leeds; M. C. W. Tate, Edinburgh; Mr. Herewig, Glasgow; Mr. Wainwright, London; Mr. T. S. Brown, Dundee; Mr. Howard Collins, Birmingham; Mr. Arthur Lucas, London; Mr. Stewart Johnson, London; Mr. Peterkin, Aberdeen; Mr. Thomas Hayes, London; the two Honorary Secretaries, and the Honorary Treasurer, with power to add to their number.

Mr. Loch seconded the list.

The representative from Northwich put in a plea for the small provincial hospitals, which he thought should be represented. He deplored that the calling of such a meeting had been necessary, as the Chancellor should have taken into his counsels the various managing bodies of the great hospitals, as he had the Friendly Societies. After a Bill had been drafted there was great difficulty in getting it amended.

Mr. Nunn (Colwyn Bay Cottage Hospital) proposed the addition of Mr. Cook, of Winsford Hospital.

The name of Mr. Alvey, of the Hospital Officers' Association, was also proposed, and a member suggested that members should convene meetings on the subject in their own districts.

Mr. Thies (Secretary Royal Free Hospital) pointed out the advantages of membership of the Association, and alluded to the work being done. The United States similar institution had a membership of 2,000, and he thought the English one should be equally strong.

The suggested committee was then approved, and the meeting closed with cordial votes of thanks to the Board of St. George's Hospital for the use of the room, and to Mr. West for presiding.

